# Efficient structured reporting in radiology using an intelligent dialogue system based on speech recognition and natural language processing

**DOI:** 10.1186/s13244-023-01392-y

**Published:** 2023-03-16

**Authors:** Tobias Jorg, Benedikt Kämpgen, Dennis Feiler, Lukas Müller, Christoph Düber, Peter Mildenberger, Florian Jungmann

**Affiliations:** 1 Department of Diagnostic and Interventional Radiology, University Medical Center of the Johannes Gutenberg-University Mainz, Langenbeckst. 1, 55131 Mainz, Germany; 2 Empolis Information Management GmbH, Kaiserslautern, Germany; 3 DFC-SYSTEMS GmbH, Munich, Germany

**Keywords:** Structured reporting, Natural language processing, Speech recognition, Dialogue system

## Abstract

**Background:**

Structured reporting (SR) is recommended in radiology, due to its advantages over free-text reporting (FTR). However, SR use is hindered by insufficient integration of speech recognition, which is well accepted among radiologists and commonly used for unstructured FTR. SR templates must be laboriously completed using a mouse and keyboard, which may explain why SR use remains limited in clinical routine, despite its advantages. Artificial intelligence and related fields, like natural language processing (NLP), offer enormous possibilities to facilitate the imaging workflow. Here, we aimed to use the potential of NLP to combine the advantages of SR and speech recognition.

**Results:**

We developed a reporting tool that uses NLP to automatically convert dictated free text into a structured report. The tool comprises a task-oriented dialogue system, which assists the radiologist by sending visual feedback if relevant findings are missed. The system was developed on top of several NLP components and speech recognition. It extracts structured content from dictated free text and uses it to complete an SR template in RadLex terms, which is displayed in its user interface. The tool was evaluated for reporting of urolithiasis CTs, as a use case. It was tested using fictitious text samples about urolithiasis, and 50 original reports of CTs from patients with urolithiasis. The NLP recognition worked well for both, with an F1 score of 0.98 (precision: 0.99; recall: 0.96) for the test with fictitious samples and an F1 score of 0.90 (precision: 0.96; recall: 0.83) for the test with original reports.

**Conclusion:**

Due to its unique ability to integrate speech into SR, this novel tool could represent a major contribution to the future of reporting.

## Key points

1. We developed an NLP-based reporting tool that converts free text into structured reports.
2. The reporting tool sends visual feedback if relevant findings are missed.
3. Testing with text samples and original free-text reports revealed excellent NLP recognition.
4. The reporting tool successfully enables the integration of speech into structured reporting.

## Background

In radiology, structured reporting (SR) is strongly recommended by professional societies [1, 2]. Compared to free-text reporting (FTR), SR has numerous advantages—including better readability, better comparability, and more detailed content of reports [3–5]. However, SR is not yet the main form of reporting in radiology, and its use remains low. This is because SR also has disadvantages. Since SR templates comprise checkboxes and drop-down menus, the radiologist’s gaze must focus on the reporting monitor to fill in the template. SR requires the use of a mouse and keyboard, rather than the speech recognition handheld that is commonly used in FTR. Besides being time-consuming, this carries the risk of distraction from the image study (*“look away”* problem) [6].

Speech recognition in reporting was introduced in the early 1980s [7]. Since then, it has gained broad acceptance among radiologists, and studies have shown its benefits, including reduced report turn-around times [8]. As rising numbers of examinations lead to steadily increasing workloads for radiologists, time-efficient reporting is crucial [9]. Improved integration of speech recognition into SR is necessary to increase the performance and acceptance of SR [10]. Artificial intelligence (AI) has gained momentum in radiology over recent years and could be a gamechanger in this task. AI can help radiologists facilitate processes in various diagnostic and non-diagnostic topics [11–13]. For example, AI tools can automatically volumetrize organs in computer tomography [14] or detect fractures in X-rays [15]. Moreover, AI is expected to have positive impacts on the radiology report generation in the future. AI algorithms might be able to automate the reporting of complex cases and reduce error rates [12].

Natural language processing (NLP) is an AI-related field that offers a machine-based approach to automatically structure and analyze free text. NLP can interpret words in sentences and understand words in context, combining methods such as linguistics, pattern matching, and machine learning. Several studies show that NLP can successfully extract content from free-text reports and automatically put it into a structured form [16–18]. Extracted content can be categorized using RadLex, which is a specialized radiologic lexicon that contains concepts with definitions and synonyms, as well as relations between them [19]. Although studies have widely explored the potential of NLP to analyze the content of existing free-text radiology reports, it has not yet been used for the creation of structured radiology reports from free text.

Dialogue systems, such as chatbots, are NLP-based conversational agents with different levels of dialogue management. There are several potential use scenarios for chatbots in radiology. Chatbots can be implemented in interactions with patients for screening purposes, as demonstrated in the COVID-19 screening of outpatients before radiological examinations [20]. Furthermore, a chatbot has been evaluated for educational purposes with gynecology patients before breast biopsies [21]. To our knowledge, there are no available data regarding the integration of any kind of dialogue system into the reporting workflow for interaction with radiologists themselves.

Combining these considerations, we developed a novel reporting tool that can automatically convert free text into a structured report using speech recognition and NLP. The tool was designed as a task-oriented dialogue system, which assists the radiologist during the reporting process. Furthermore, we evaluated this tool in the use case of abdominal CT in patients with suspected urolithiasis.

## Methods

The novel reporting tool was created by combining preexisting and newly developed components. The user interface was developed as a Microsoft^®^.NET-application. For speech recognition, *indicda*^*®*^* speech* (DFC-Systems GmbH, Munich, Germany) was used. The task-oriented dialogue system was built on top of *Empolis Knowledge Express*^®^ (Empolis Information Management, Kaiserslautern, Germany).

**Fig. 1:**
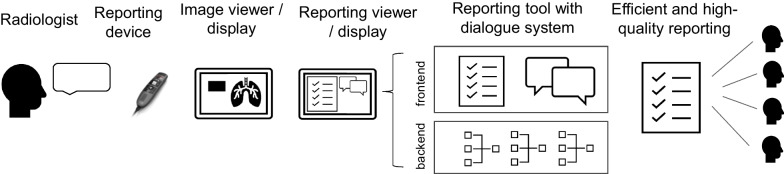
Graphic representation of the reporting workflow using the reporting tool. Reporting is done as free text using speech recognition. The reporting tool comprises a user interface (frontend), and a dialogue system that enables NLP-based conversion of free text into a structured report and communication with the radiologist (backend). The combination of these components enables efficient and high-quality reporting

The workflow using the reporting tool is similar to a standard reporting workflow in clinical routine. The radiologist uses a speech recognition handheld for FTR. Two monitors are needed to display the user interface and the imaging study in a PACS viewer. The tool comprises a frontend represented by the user interface, and a backend represented by technical features—including speech recognition, the NLP components, and the task-oriented dialogue system (Fig. 1).

The tool was designed to be applicable for the reporting of any radiological examination. Abdominal CT in suspected urolithiasis was selected as the first use case. Therefore, we applied a standard urolithiasis SR template that is used in clinical routine at our institution. Urolithiasis was chosen because it is a common examination in radiologists’ daily work and is adequately complex to test and evaluate the tool.

### Frontend

**Fig. 2:**
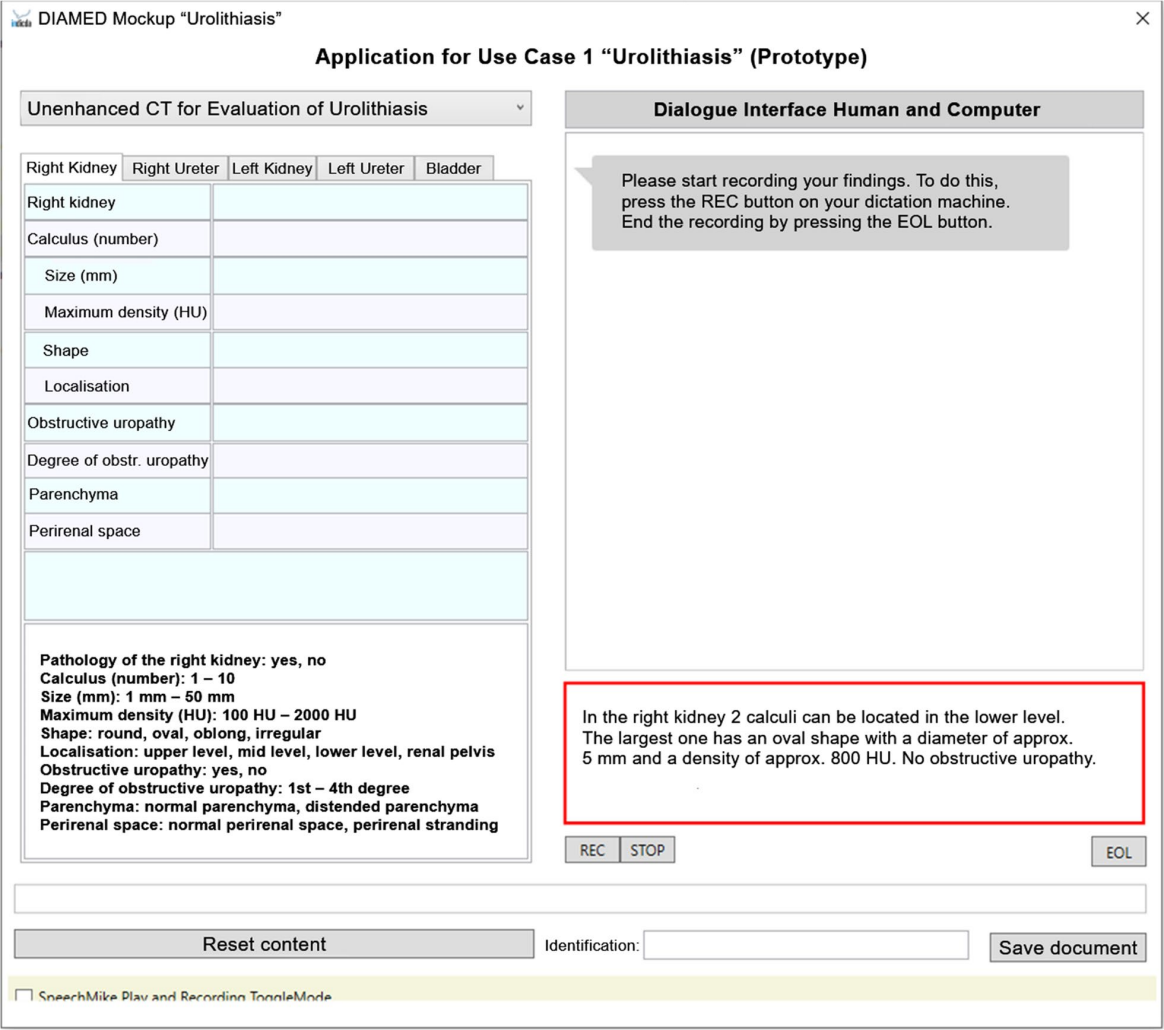
Screenshot of the reporting tool’s graphical user interface (translated from German to English language). On the left side, the to-be-completed SR template is shown, including content suggestions at the bottom. On the right side, the reporting window (bottom) and the dialogue window (top) are shown. The radiologist has started reporting in a free-text form using speech recognition

**Fig. 3:**
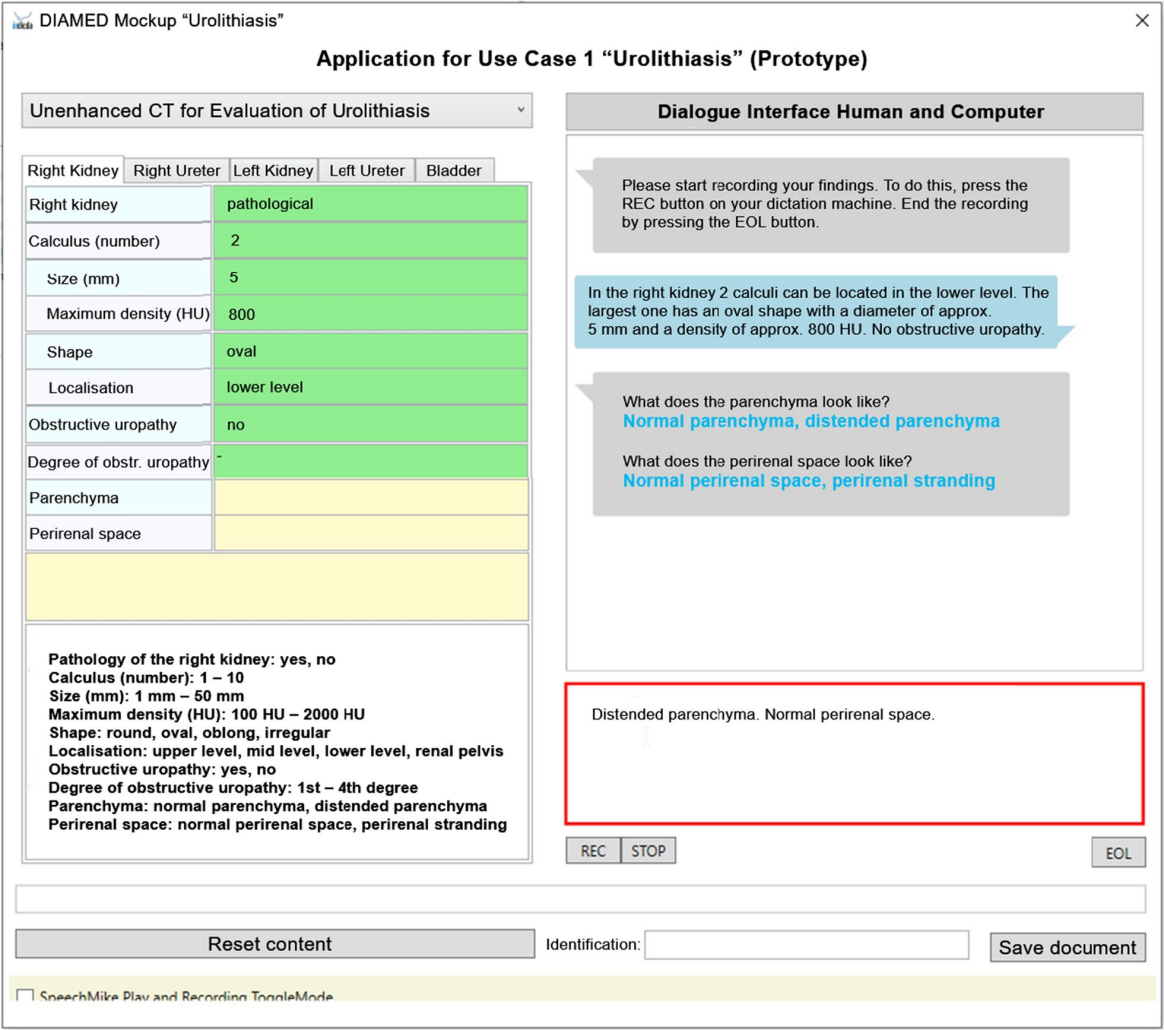
Translated screenshot of the reporting tool’s graphical interface, further along in the reporting process. The previously dictated free text regarding nephrolithiasis of the right kidney (shown in Fig. 2) has been transferred to the SR template on the left side (green background). Since no statements were made regarding the parenchyma and perirenal space, the dialogue system advises the radiologist to discuss these findings (dialogue window). The radiologist can start a new turn by dictating free text about the parenchyma and perirenal space (reporting window)

The user interface comprises two parts (Fig. 2). The first part is a template window on the left side, which shows the to-be-completed SR template for the use case, including an overview of possible response options at the bottom. The SR template separately considers individual organ systems, e.g., right kidney, right ureter, left kidney, left ureter, and urinary bladder. In the upper left margin, there is a tab for each organ, and the radiologist must report on each organ consecutively. The second part comprises a reporting window and a chat window on the right side. Radiologists can activate their speech recognition handheld and dictate a free-text report that appears in the reporting window on the bottom. A red margin of the reporting window indicates that speech recognition is activated. Upon finishing the report, the SR template on the left side is automatically filled in. If findings are missing, the dialogue system sends the radiologist messages that appear in the chat window at the upper right part of the interface (Fig. 3). Upon completion of an organ system, the interface automatically switches to the next. If the radiologist mentions *“no pathologies”* for a system, it is automatically completed and the system switches to the next. A yellow/green bar on the bottom of the interface displays the progress of reporting.

### Backend

**Fig. 4:**
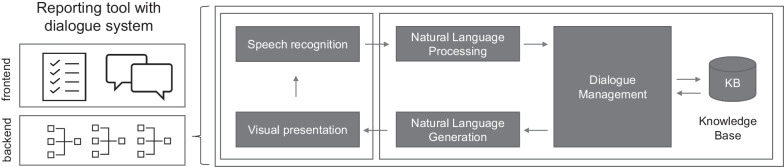
Graphic representation of the reporting tool’s backend. The dialogue system comprises speech recognition, natural language processing (NLP), dialogue management with a knowledgebase, natural language generation (NLG), and visual presentation. The arrows represent the typical data flow. Speech recognition and visual presentation were built on top of *indicda*^*®*^* speech*. NLP, dialogue management, knowledgebase, and NLG were built on top of *Empolis Knowledge Express*^*®*^

The backend portion of the reporting tool comprises a task-oriented dialogue system with several technical components, including speech recognition, NLP, dialogue management, knowledgebase, natural language generation (NLG), and audiovisual representation (Fig. 4) [22]. It was designed to help radiologists document their findings as efficiently as possible. Therefore, it fills in the template on behalf of the radiologist. It was designed as a stateless (web) service, which means that with the same input, the output will be the same, independent of any previous inputs. Its input is communication by the user. As output, it returns the completed template and additional advice for the user. The system has to handle various challenges with spoken language understanding, e.g., synonyms, abbreviations, negations, speculations, and uncertain information.

The systems’ components interact with each other (Fig. 4). Speech input is transformed to unstructured text by speech recognition, and the NLP component translates the text into a structured form (segment, concept, and negation detection). For negation detection, a neural network probabilistically determines the negation status and selects the most probable status (affirmed, negated, or speculated). For segment and concept detection, the NLP component applies rule-based matching approaches. RadLex concepts are assigned to the structured content. Then, the dialogue management takes over the structured content, recognizing and returning the user’s intents based on the structured content delivered. Detected intents are used to complete the fields of the SR template (template intents), and to advise the user of possible considerations (advice intents). For this process, the dialogue management uses a knowledgebase containing the necessary expert knowledge on intents. Data are further transferred to the NLG component, which transforms intents from machine-generated codes into a human-understandable form. A visual presentation component communicates the results to the user by automatically completing the SR template, and through the chat window on the user interface. After the visual presentation, a dialogue turn is completed. A new turn can be initiated by the user.

**Fig. 5:**
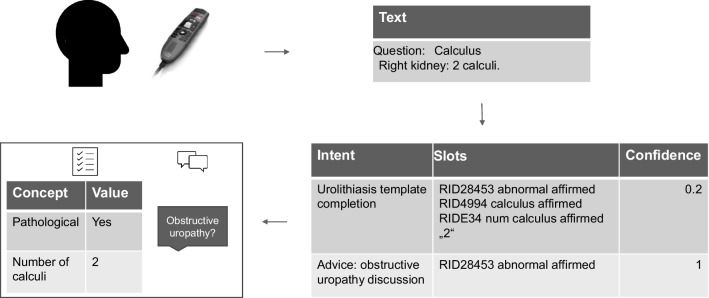
Functionality of the dialogue system. Text is translated into structured content of intents and slots. The template is completed using RadLex terms. Advice intents are returned to the user. A confidence is computed for each intent

Figure 5 shows an example of the reporting workflow. The user has reported two calculi in the right kidney. Speech is translated to text (speech recognition), and text is translated into structured content of intents and slots (NLP and dialogue management). The detected intent is the completion of the template. Slots are the values of fields for the SR template. Structured content is used to complete the template, and to give the user natural language advice in the graphical user interface (NLG and visual presentation). The template is completed in RadLex terms (RID28453 abnormal and RID4994 calculus). Additional advice intents are returned to the user. In this case, the user is advised to discuss a potential obstructive uropathy (RID34394 obstructive uropathy). Every intent has a confidence. For template intents, confidence equals the template’s completeness, which can be visualized on the progress bar of the user interface. For advice intents, the system uses the confidence to filter out unlikely intents. Besides standard RadLex concepts, for some codes the actual text is used to complete the template—for example, in this case, the number of calculi is entered as “two.” In such cases, the text may be preprocessed by the visual presentation component to fit the template.

For proper functionality of the system with the use case, special configurations were needed for single components. Since RadLex does not contain all the concepts needed for the urolithiasis template, five concepts had to be added. These were labeled as RadLex ID extensions (RIDE). Synonyms for all concepts were added.

A set of 85 rules for the knowledgebase determines the behavior of the dialogue management in intent detection. These rules define which SR template fields are needed in a specific case—meaning that if a calculus is documented, the user will be asked for additional information about its features (size, density, morphology, and location). Further rules define relations between values, enabling implicit completion of SR template fields (e.g., if a calculus is documented, the kidney is automatically documented as abnormal). The rules are described as decision tables in an excel sheet, which are automatically transformed to the format used in the knowledgebase [23]. The knowledgebase uses an ontology language (OWL/RDF) and a reasoner (ELK reasoner).

The NLG component uses a set of 57 mappings between advice intents and natural language questions for the chat window. It also uses mappings between the SR template and its natural language representation with 65 variables.

## Results

The reporting tool was tested for its ability to convert free text into a structured report in two ways. First, we evaluated the tool using fictitious text examples for urolithiasis, which were created by a board-certified radiologist. The fictitious examples were realistic but not related to any specific patient. The urolithiasis template has 41 fields. Each field was tested twice, yielding a total of 82 fictitious examples. The results of the test case showed an F1 score of 0.98 (precision 0.99; recall 0.96).

False-positive results were caused by ambiguous descriptions of morphology—for example, if a morphologic descriptor did not relate to the morphology of a calculus but rather to a parenchyma defect. False-negative results were caused by missing synonyms of parenchyma descriptors, perirenal space descriptors, and grade descriptors.

Secondly, we evaluated the reporting tool using 50 original free-text reports from patients who underwent unenhanced CT for suspected urolithiasis. This evaluation was done exemplarily for the right kidney; therefore, we only included reports that mentioned at least one calculus in the right kidney. These reports were manually selected from our imaging archive and were authored by 13 different board-certified radiologists. Free text regarding the right kidney was manually extracted from the reports. The reports were gold standard labeled by manually recording the values for the right kidney from the tools’ SR template in an excel sheet—including the number of calculi, size, density, morphology and location of the largest calculus, obstructive uropathy, and grade of uropathy. The text from each report was automatically analyzed by the reporting tool and compared to the gold standard. A total of 350 values (50 cases with 7 fields per case) were compared to each other. The total number of possible true positives was 237. The test case showed an F1 score of 0.90 (precision 0.96; recall 0.83).

False-positive results were caused by text examples that did not relate to the right kidney but rather to other segments, like the left kidney. Moreover, three-dimensional size descriptions for calculi were not correctly detected, since the SR template only offers one-dimensional size specifications. False-negative results occurred for the field *number of calculi* in reports that mentioned a single renal calculus and referred to it only as “calculus” instead of explicitly naming it “one calculus.”

## Discussion

Our reporting tool can automatically convert free-text reports into structured reports and send messages concerning missing findings. To our knowledge, this is the first approach to use NLP for the generation of structured reports, as well as the first approach to integrate a dialogue system into the reporting process.

The feasibility of this tool was proven by the high test scores obtained using fictitious text samples and existing reports from our archive (F1 scores: 0.98 and 0.90). Other studies of NLP-based extraction of structured content from free-text reports have shown comparable or slightly lower scores. For example, an NLP engine comparable to ours achieved an F1 score of 0.81 for fracture detection from free-text X-ray reports [17]. In a systematic review of 164 publications regarding the application of NLP to radiology reports, 83% of the studies reported an F1 score of < 0.85. However, most of these approaches have used data sets of different complexity, making it difficult to comparatively evaluate them [24].

Our present test focused on evaluation of the right kidney, exemplarily on behalf of the whole template. This was a legitimate approach since each organ system is separately reported in our reporting tool. Therefore, evaluation of the other organ systems (ureters, bladder, and left kidney) should not show important deviations. Furthermore, all test cases were positive for nephrolithiasis of the right kidney. No negative reports were used for testing.

In most previous studies, NLP has been retrospectively applied on existing radiology reports for structured content extraction [16–18]. Such retrospective study design carries the risk of model over-fitting and also focuses on automated data analysis rather than real-world clinical application of NLP [24]. Our present approach is unique, since it enables the prospective use of NLP for the creation of structured reports.

The creation of structured reports from free-form dictation was described as *the ultimate quest* at the beginning of this century, when SR was first evolving. Proposals have been made for ideal reporting systems combining both features, which are considered necessary for future reporting [10]. However, no working system was developed, which may partly explain why SR has not yet become the main reporting form. Since the early 2000s, there have been massive advances in the field of AI, which offer huge potential to facilitate and automate processes in the diagnostic imaging workflow [13]. The possibilities offered by NLP are sufficiently advanced to make it feasible to develop the desired reporting system.

The successful implementation of speech into SR may enable us to solve the so-called look away problem of SR [6]. As imaging data sets become increasingly large and complex, most of the radiologist’s cognitive effort should be focused on the images, rather than on complicated reporting templates that require the use of a mouse and keyboard for completion. With our tool, radiologists can continue to rely on the speech recognition that they have been conveniently using for decades, while also benefiting from the enormous proven advantages of SR, like the possibility of datamining, facilitation of epidemiologic research, higher referrer satisfaction, etc. [25–27]. Our novel approach merges the advantages of SR and free-text reporting, while avoiding their shortcomings.

Chatbots have been implemented in several radiology departments for patient screening and patient education purposes [20, 21]. However, there are no published data regarding their use in the reporting workflow. In our reporting tool, the dialogue system serves as intelligent decision support for the radiologist. Since it can also complete an SR template, its capabilities go beyond that of a normal chatbot. Feedback from the dialogue system about missing findings may lead to higher completeness, and thus a higher quality of reports. This can be especially helpful for inexperienced radiology residents. In fact, the system can guide them through the report, providing a learning effect.

Beyond the urolithiasis use case presented here, this novel reporting tool can easily be adapted for the reporting of any other radiological examinations for which SR templates are available or can be created. Many professional societies offer freely available SR templates online [5]. As of December 2022, the *RSNA* RadReport website alone offers over 400 SR templates [28]. This availability of large numbers of preexisting templates underlines the possible broad applicability of our tool. We are currently integrating additional examinations into our tool, including MRI for rectal cancer staging and MRI of the lower spine in suspected disk herniation, with more to follow.

### Strengths, weaknesses, and limitations

As summarized from the above, the main strengths of our tool are its abilities to automatically create structured reports from free-form dictation, and to send visual feedback to the radiologist concerning missing findings. However, it also has several weaknesses and limitations. First, we developed the tool for use in a German-speaking area. Therefore, the SR template was developed in the German language, and the NLP engine was trained using German free-text reports. However, since the system uses RadLex concepts for categorization of findings, it would also be easily applicable to the English language. Second, the tool is not yet implemented into the clinical imaging workflow. At our institution, in 2016, we established an IHE-MRRT-compliant web-based platform for conventional SR, which is connected to the RIS [29]. The best way to integrate our tool would likely be to establish a connection (e.g., FHIR) between the tool and the SR reporting platform. This would enable the tool to provide support in completing templates on the platform, without changing the connection between the platform and RIS.

There remains a need for additional data regarding how radiologists experience and approve our reporting tool in actual clinical practice. The evaluation of user experience is currently in progress, but goes beyond the scope of the present paper, which focuses on technical development and validation.

Future studies will evaluate the ease of use and the possible need to train radiologists on how to use the tool. Additionally, the tool’s value will be investigated in terms of its potential to avoid errors in reporting, and to monitor radiologists’ performance. In a user study, the reporting time and the completeness of reports using the reporting tool will be compared to those with conventional structured reporting. Lastly, the tool may have a positive effect on the availability of usable data for AI development, as it provides large datasets for training and validation in a highly structured form.

## Conclusion

Our reporting tool is unique in its ability to automatically convert free-text reports into structured reports, and to send the radiologist messages about missing findings. Through the NLP-based integration of speech into SR, it combines the advantages of SR and FTR, while avoiding the shortcomings of both methods. Its use in clinical routine may enable the creation of structured and complete reports that could conveniently be freely dictated via speech recognition. Therefore, this novel tool has great potential to revolutionize the future of radiology reporting.

## Data Availability

The datasets generated and/or analyzed during the current study are available from the corresponding author on reasonable request.

## Abbreviations

AI: Artificial intelligence
FTR: Free-text reporting
NLG: Natural language generation
NLP: Natural language processing
SR: Structured reporting
